# Clinicopathological significance of indoleamine 2,3-dioxygenase 1 expression in colorectal cancer

**DOI:** 10.1038/bjc.2011.513

**Published:** 2011-11-22

**Authors:** L Ferdinande, C Decaestecker, L Verset, A Mathieu, X Moles Lopez, A-M Negulescu, T Van Maerken, I Salmon, C A Cuvelier, P Demetter

**Affiliations:** 1 Department of Pathology, Ghent University Hospital, Universiteit Gent, Block A, 5th floor, De Pintelaan 185, B-9000 Ghent, Belgium; 2 Laboratory of Image Analysis and Synthesis, Université Libre de Bruxelles, Brussels, Belgium; 3 Department of Pathology, Erasme University Hospital, Université Libre de Bruxelles, Brussels, Belgium; 4 Department of Clinical Chemistry, Microbiology and Immunology, Ghent University Hospital, Universiteit Gent, Ghent, Belgium

**Keywords:** indoleamine 2,3-dioxygenase 1, colorectal cancer, immunohistochemistry, tumour invasion front, colon cancer cell lines

## Abstract

**Background:**

Indoleamine 2,3-dioxygenase 1 (IDO1) is a tryptophan-catabolising enzyme that induces immune tolerance by modulating T-cell responses. Carcinomas may create an immunosuppressive state via IDO1 expression. Here we examined a possible contribution of IDO1 on this phenomenon and investigated whether IDO1 has prognostic value in colorectal cancer (CRC).

**Methods:**

IDO1 expression was investigated by quantitative PCR and western blotting in three colon cancer cell lines, in basal state and after interferon (IFN)-*γ* stimulation. Semi-quantitative immunohistochemistry was used to evaluate IDO1 expression in 265 pT1-4N0-2Mx-staged CRCs. Results were related to clinical variables and correlated with amounts of CD3^+^ and CD8^+^ T lymphocytes, which were quantitatively evaluated using image analysis.

**Results:**

*In vitro* expression of IDO1 depended on IFN-*γ* stimulation. Higher IDO1 expression at the tumour invasion front was an independent adverse prognostic factor in pT1-4N1Mx-staged CRC. It was associated with overall survival (*P*=0.001) and with metachronous metastases (*P*=0.018). IDO1 expression was not associated with the presence of CD3^+^ or CD8^+^ T lymphocytes.

**Conclusion:**

Higher IDO1 expression at the tumour invasion front is involved in CRC progression and correlates with impaired clinical outcome, suggesting that IDO1 is an independent prognostic indicator for CRC.

The incidence of colorectal cancer (CRC) is estimated at one million new cases per year worldwide and this has continued to increase over the last 25 years (Jemal *et al*, 2009; West *et al*, 2009). Although the overall 5-year survival from CRC is approximately 60% in population-based series, prognosis is well established to be strongly linked to a stage at presentation. However, there is variation in the outlook for patients with the same-stage disease; hence, it is highly desirable to have additional markers more strictly related to the intrinsic behaviour of the CRC to better define the clinical approach and individualise the therapy. This is particularly important for those patients with lymph node metastases, in whom active management is preferred when no contraindications due to comorbidity or age exist. New prognostic markers may reveal the heterogeneity in patients with the same-stage CRC and guide the individualised therapy, which will further improve the survival of patients with CRC, as well as prevent the unnecessary use of adjuvant treatment.

Indoleamine 2,3-dioxygenase 1 (IDO1) is a heme-containing intracellular enzyme that catalyses the initial and rate-limiting step in the catabolism of the essential amino acid tryptophan along the kynurenine pathway (Takikawa *et al*, 1986). The role of IDO1 in tumour-induced tolerance was first described by Uyttenhove *et al* (2003) in a murine model, in which they showed that IDO1 expression in cancer cells protects tumours from attack by tumour-associated antigen-specific host cytotoxic T cells. Tryptophan depletion and accumulation of immunomodulatory tryptophan metabolites contribute to the immunosuppressive capacities of IDO1, which will specifically affect T-cell metabolism and function (Moffett and Namboodiri, 2003; Fallarino *et al*, 2006). The relationship between IDO1-expressing tumours and T lymphocytes is, however, complex, as interferon-*γ* (IFN-*γ*), produced mainly by T lymphocytes in response to various immune stimuli (Farrar and Schreiber, 1993), is a major inducer of IDO1 (Takikawa *et al*, 1999; Hwu *et al*, 2000).

With regard to CRC, data on IDO1 are scarce. Although IDO1 expression in cancer cell lines has been claimed to be constitutive (Uyttenhove *et al*, 2003), others have reported that the expression in colon cancer cell lines is dependent on IFN-*γ* (Brandacher *et al*, 2006). The same group showed that high IDO1 immunoreactivity in CRC correlated significantly with the frequency of liver metastases (Brandacher *et al*, 2006). Seventy-one percent of patients with IDO1-low primary tumours were free of metastasis, whereas this was the case for only 50% of patients with IDO1-high primary tumours. Kaplan–Meier analysis showed the crossing of survival curves at 45 months, and therefore, they could not show any survival advantage. However, high IDO1 expression emerged as an independent prognostic variable. In a small subset of samples, double immunostainings for IDO1 and CD3 were performed, revealing a significantly higher proportion of intratumoural CD3^+^ cells in IDO1-low expressing tumours as compared with IDO1-high expressing tissue samples. Another research group could not confirm the correlation between IDO1 expression in CRC cells and survival, but these authors demonstrated that high density of IDO1-expressing cells in tumour-draining lymph nodes without metastasis was associated with a lower survival rate (Gao *et al*, 2009).

The present study aimed to further investigate a possible prognostic role of IDO1 expression in a large series of CRC cases, without evidence of distant metastases at time of presentation, and to study the relationship between IDO1 expression in tumour cells and the presence of lymphocytes in these tumours.

## Materials and methods

### Cell lines and culture

Three human colon carcinoma cell lines, namely Caco-2, HT-29 and T84 were obtained from the American Type Tissue Collection (Manassas, VA, USA; ATCC HTB-37, HTB-38 and CCL-248, respectively). Caco-2 cells were maintained in Dulbecco's modified Eagle's medium (DMEM; Gibco, Merelbeke, Belgium) enriched with non-essential amino acids (Gibco), HT-29 cells in DMEM with L-glutamine and pyruvate (Gibco), and T84 cells in DMEM : F12 (1 : 1; Gibco). All media were supplemented with 10% (for Caco-2 and HT-29) or 5% (for T84) heat-inactivated fetal calf serum (Gibco), and with 1% antibiotics and antimycotics (Gibco). The cells were cultured at 37 °C in humidified air containing 5% CO_2_. Before stimulation with IFN-*γ*, cells were grown in serum-free medium for 5 h. Then, they were grown for 48 h in medium without fetal calf serum, and with or without IFN-*γ* (1000 U ml^−1^, Sigma-Aldrich, Bornem, Belgium).

### Real-time quantitative reverse transcription-PCR

Total RNA was extracted from the three cell lines (stimulated with IFN-*γ* and non-stimulated) by using an RNeasy Mini Kit (Qiagen, Hilden, Germany), with RNase-free DNase I treatment on column. First-strand cDNA was synthesised from 2 *μ*g of total RNA with an iScript cDNA synthesis kit (Bio-Rad, Hercules, CA, USA). For real-time quantitative reverse transcription-PCR (RT–PCR), a SYBR Green I assay was used. Reactions containing primers for *IDO1* (*IDO1* forward: 5′-CTACCATCTGCAAATCGTGACTAAGT-3′, *IDO1* reverse: 5′-GAAGGGTCTTCAGAGGTCTTATTCTC-3′) and three reference genes (*SDHA, UBC, YWHAZ*, primer sequences: see http://medgen.ugent.be/rtprimerdb/; Pattyn *et al*, 2006), were run in duplicate on an iQ5 real-time PCR detection system (Bio-Rad). Gene expression levels were calculated by using qBase analysis software (http://www.biogazelle.com) (Hellemans *et al*, 2007).

### Western blotting

Cells were washed with ice-cold PBS and cell lysates were prepared using lysis buffer (1% Triton-X-100/1% NP-40/PBS) containing protease inhibitor cocktail tablets (Roche, Vilvoorde, Belgium). Western blotting was performed for evaluating protein expression of IDO1 and phosphorylated STAT1 (pSTAT1). The latter was carried out to confirm the IFN-*γ* stimulation, as STAT1 is a downstream molecule in the signal transduction pathway of IFN-*γ* that is phosphorylated after binding of the IFN-*γ* to its receptor. Electrophoresis on 4–12% Bis-Tris gels with 3-(N-morpholino)propanesulfonic acid running buffer (Invitrogen, Paisley, UK) was performed in an XCell II Mini-Cell electrophoresis unit (Invitrogen). The proteins were blotted onto a nitrocellulose membrane, which was blocked in 10% milk powder/0.1% Triton-X-100/PBS (for IDO1) or in 5% milk powder/0.1% Tween-20/TBS (for pSTAT1). Subsequently, the membranes were incubated with the primary antibodies (IDO1: dilution 1 : 200, Santa Cruz Biotechnology, Santa Cruz, CA, USA; pSTAT1: dilution 1 : 200, Santa Cruz Biotechnology), followed by horseradish peroxidase-conjugated secondary antibodies (anti-rabbit IgG for IDO1 (Santa Cruz Biotechnology); anti-goat IgG for pSTAT1 (Santa Cruz Biotechnology)). Immunoreactive proteins were visualised using the biochemiluminescence technique and Hyperfilm ECL (GE Healthcare, Uppsala, Sweden) development. To confirm the loading of equal amounts of protein, membranes were stripped and reprobed with antibodies for *β*-actin (Santa Cruz Biotechnology).

### Patients

We retrospectively analysed tumour samples from 265 consecutive patients who underwent surgical resection of a primary CRC, without evidence of distant metastasis at the time of surgery. All patients underwent surgical resection at the Erasme University Hospital (Brussels, Belgium) between May 1990 and December 2000. Follow-up was available until August 2009. The study was approved by the local ethics committee.

Basic patient demographic data are summarised in Table 1. Mismatch repair (MMR) protein expression was detected by immunohistochemistry, and divided into ‘intact’ and ‘defective’ groups. Intact MMR protein expression was defined by nuclear expression of MLH1, MSH2, MSH6 and PMS2 in neoplastic epithelial cells. If stromal cells were negative for at least one of these markers, the case was considered non evaluable for MMR protein expression.

### Tissue microarray (TMA) construction

TMA blocks were constructed as described previously (Decaestecker *et al*, 2009), with a manual microarrayer (Beecher Instruments, Sun Prairie, WI, USA) to include six cores (600-*μ*m diameter) from each CRC case. Three cores were obtained from the central part of the tumour, and three cores from the invasion front.

### Immunohistochemistry

Standard immunohistochemistry was applied to 5-*μ*m thick sections to display IDO1, CD3 or CD8 expression, using specific antibodies provided by Abcam (Cambridge, UK; anti-IDO1, dilution 1 : 1000), Menarini (Florence, Italy; anti-CD3, dilution 1 : 100) or DAKO Cytomation (Glostrup, Denmark; anti-CD8, dilution 1 : 200), as detailed elsewhere (Ferdinande *et al*, 2008; Decaestecker *et al*, 2009). Briefly, the immunohistochemical expression was visualised by means of biotinylated anti-sheep IgG (Santa Cruz) and streptavidin (DAKO; for IDO1 immunohistochemistry) or streptavidin–biotin–peroxidase complex kit reagents from BioGenex (San Ramon, CA, USA; for CD3 and CD8 immunohistochemistry), with 3-amino-9-ethylcarbazole (for IDO1) or diaminobenzidine (for CD3 and CD8) as the chromogenic substrate. The sections were counterstained with hematoxylin. Negative controls consisted of replacing the primary antibodies with irrelevant immunoglobulins (Santa Cruz Biotechnology for IDO1, DAKO for CD3 and CD8).

A final validation stage was conducted by a pathologist (LF) who used Spot Browser V2E (Alphelys, Plaisir, France) on a BX50 microscope (Olympus, Aartselaar, Belgium) for a visual evaluation of the immunostained TMA slides, aiming to confirm the diagnostic and the immunostaining compliance. Only the cores satisfying all the control steps were submitted for staining evaluation.

### Evaluation of immunohistochemical stainings

**Figure 1:**
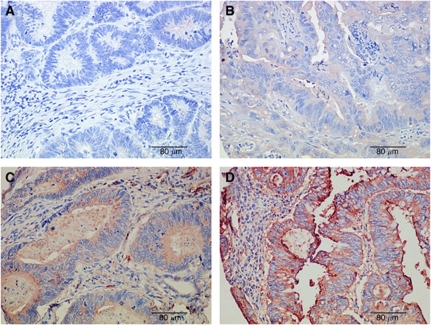
Semi-quantitative scoring system of IDO1 immunohistochemistry: (**A**) 0: no expression; (**B**) 1: weak expression; (**C**) 2: moderate expression; (**D**) 3: strong expression.

Spot Browser was also used for TMA core image acquisition and semi-quantitative and quantitative staining evaluation, using standardised protocols detailed elsewhere (Decaestecker *et al*, 2009). This evaluation was performed for each valid TMA core and pooled per patient by distinguishing central tumour part from invasion front. IDO1 expression was observed in neoplastic epithelial cells, mononuclear cells in the stroma and endothelial cells, as previously described (Ferdinande *et al*, 2008). The aim of this study was to correlate IDO1 expression in neoplastic epithelial cells with clinicopathological variables and infiltrating T lymphocytes; therefore, only IDO1 staining in neoplastic epithelium was analysed. IDO1 expression by neoplastic epithelial cells was scored semi-quantitatively on a 4-point scale (0: no expression, 1: weak expression, 2: moderate expression, 3: strong expression) by a pathologist (LF; Figure 1). The mean score per tissue region was computed for each patient. The presence of CD3^+^ and CD8^+^ T lymphocytes were quantified by means of standardised image analysis and reported as the labelling index, which is the percentage of the immunostained tissue area computed in the reference tissue region (central tumour part or invasion front), as detailed elsewhere (Decaestecker *et al*, 2009).

### Statistical analysis

The Kruskal–Wallis test was used to compare independent groups of numerical data (e.g., across tumour stages). When this multi-group test was significant, *post-hoc* tests (Dunn's procedure) were used to compare the group pairs of interest, thus avoiding multiple comparison effects. The comparison of quantitative expression levels between central tumour part and invasion front (i.e., dependent samples) was carried out by means of the Wilcoxon matched pair test. The Spearman's correlation test was used to analyse non-parametric correlation between staining features. Finally, survival data were analysed using the standard Kaplan–Meier analysis and the multivariate Cox regression. Survival curves were compared using the log-rank test. All the statistical analyses were carried out using Statistica (Statsoft, Tulsa, OK, USA).

## Results

### IDO1 expression in colon cancer cell lines

**Figure 2:**
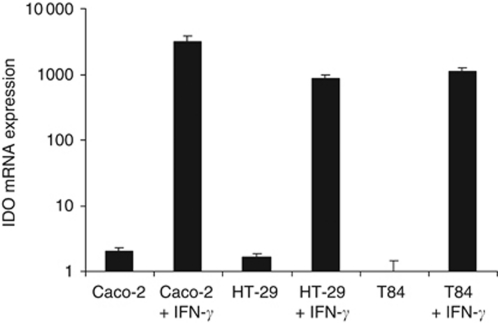
Relative mRNA expression level of *IDO1* in non-stimulated and IFN-*γ* stimulated Caco-2, HT-29 and T84 cells. The *IDO1* expression level in the sample with the lowest expression (non-stimulated T84 cells) was set to 1. Columns, mean of two different RT–PCR analyses; bars, s.e.m.

**Figure 3:**
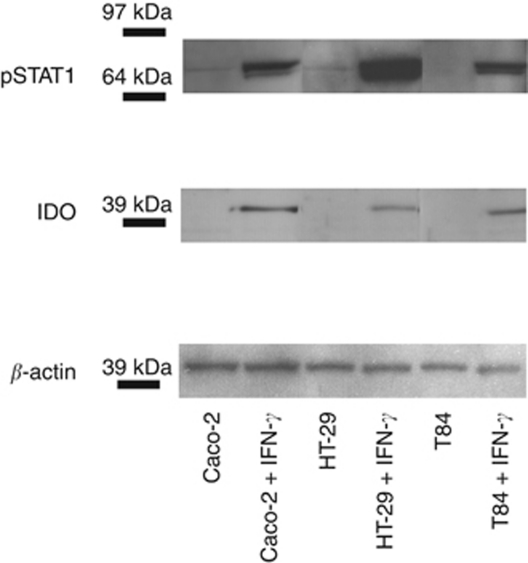
Western blot analysis of pSTAT1 and IDO1 protein expression in non-stimulated and IFN-*γ*-stimulated Caco-2, HT-29 and T84 cells. In stimulated cell lines, an 84- and 91-kDa double pSTAT1 band and a 42-kDa IDO1 band were visualised.

Figure 2 displays the quantitative RT–PCR results, showing the relative gene expression levels of *IDO1* in the three colon carcinoma cell lines under non-stimulated and stimulated conditions. Basal mRNA expression levels of *IDO1* were very low in non-stimulated cells, but *IDO1* expression was strongly induced by IFN-*γ*. These results were confirmed at the protein level using western blot analysis. In none of the tumour cell lines, IDO1 protein expression was visualised without IFN-*γ* stimulation. Protein extracts from IFN-*γ*-treated cells revealed both expression of pSTAT1, indicative of successful IFN-*γ* stimulation, and expression of IDO1 (Figure 3).

### IDO1 expression in CRCs

Varying degrees of IDO1 expression by neoplastic epithelial cells were detectable in 239 out of 265 cases (90%). In 191 cases (72%), epithelial IDO1 positivity was found in both the invasion front and the centre of the tumour. A total of 13 cases (5%) were IDO1 positive in the invasion front only; 30 cases (11%) were IDO1 positive in the tumour centre only. In five cases, IDO1 positivity was present in the invasion front, whereas the centre could not be evaluated by loss of material.

Both the IDO1 expression in the invasion front and in the centre of the tumour did not vary significantly across tumour stages (data not shown).

### Presence of CD3^+^ and CD8^+^ T lymphocytes in CRCs

Both CD3- and CD8-labelling index were significantly higher in the invasion front than in the centre of the tumour (*P*<10^−6^), but did not significantly vary across tumour stages (data not shown).

### Prognostic evaluation of IDO1 expression

**Figure 4:**
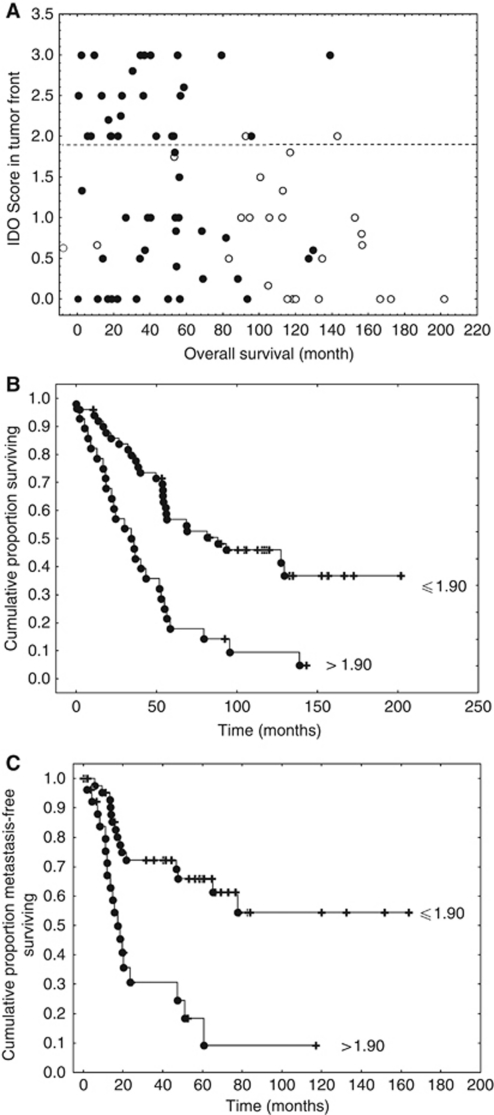
Prognostic value of IDO1 scores at the tumour invasion front. (**A**) Determination of a score threshold identifying short survival patients (mean score >1.9), where open/black dots identify alive/dead patients. (**B**) Overall survival curves (*P*=0.0004); (**C**) metastasis-free survival curves (*P*=0.0003; log-rank test). Complete and censured data are shown by dots and crosses, respectively.

Using multivariate survival analyses, we first identified the clinicopathological variables, with impact on prognosis in the complete case series of 265 CRC patients. The results showed that age, T stages and N stages are independent negative prognostic factors for overall survival, and male gender, T stages and N stages for the development of metachronous metastases (data not shown). Taking these factors into account, we identified higher IDO1 expression at the tumour invasion front as being an independent negative prognostic factor for pT1-4N1Mx-staged CRC (Table 2). Although in this subgroup T stage was no longer an independent prognostic factor, tumoural IDO1 expression at the invasion front was significantly associated with overall survival (*P*=0.001, Table 2A) and with the development of metachronous metastases (*P*=0.018, Table 2B). By analysing the distribution of the IDO1 scores at the tumour invasion front, we found that a large majority of patients with a mean score higher than 1.9 had a short survival (Figure 4A). The Kaplan–Meier curves shown in Figures 4B and C confirm the prognostic value of this IDO1 threshold in terms of overall and metastasis-free survival.

In contrast, IDO1 expression in the central part of the tumour did not exhibit any prognostic value or correlation with IDO1 expression at the tumour invasion front. In fact, about half of the cases presented higher expression levels in the central part, whereas the other half exhibited higher levels at the invasion front (data not shown).

As in the pT1-4N1Mx subgroup only three tumours showed defective MMR protein expression, no statistical analysis was conducted in search for a possible correlation between IDO1 and MMR protein expression.

### Association of IDO1 expression with the presence of T lymphocytes

As IDO1 function (resulting in tryptophan depletion and accumulation of metabolites) influences T-cell metabolism, we investigated whether the negative prognostic effect of IDO1 expression at the tumour invasion front in pT1-4N1Mx-staged CRC was associated with a decrease in the number of CD3^+^ or CD8^+^ T lymphocytes. Spearman's correlation analysis however did not reveal any significant correlation between IDO1 score and CD3- or CD8-labelling index (data not shown).

## Discussion

In this study, we demonstrated that higher IDO1 expression at the tumour invasion front correlates with progressive disease and impaired clinical outcome in a specific subset of patients with CRC, suggesting that IDO1 is an independent and reliable prognostic indicator for CRC. These data confirm, extend and specify an earlier investigation in which a prognostic value of IDO1 in CRC was detected (Brandacher *et al*, 2006). As this is an immunohistochemical study, it is not possible to determine whether high IDO protein expression is associated with high IDO1 functional activity or is mainly the result of nonspecific deregulation of gene expression as a characteristic of a more aggressive tumoural phenotype. But, as our observations identify IDO expression as a prognostic marker, it is tempting to speculate about the role of IDO1 in tumour immune escape.

Invasion of tumour cells depends on a permissive host environment at the invasive site of the primary tumour, as well as at the site of metastasis. Likewise, the tumour cells of the invasion front may display features, which differ from those in the inner parts of the tumour. For example, downstream targets of the Wnt/*β*-catenin pathway and *β*-catenin itself exhibit stronger protein expression at the invasion front of colorectal tumours (Baldus *et al*, 2004; Hiendlmeyer *et al*, 2004; Beiter *et al*, 2005; Gavert *et al*, 2005). The prognostic value of IDO1 expression at the invasion front, but not in the central part of the tumour, further indicates that the invasion front indeed constitutes a biologically defined compartment. In this specific area, IDO1 can interact with tumour-infiltrating lymphocytes (Laghi *et al*, 2009). But, whether IDO1 activity is able to affect the amount of local tumour-infiltrating lymphocytes, which is an independent prognostic factor in patients with CRC (Waldner *et al*, 2006; Laghi *et al*, 2009), remains controversial. Although some studies are in favour of this hypothesis (Brandacher *et al*, 2006; Inaba *et al*, 2009), functional inactivation of effector CD8^+^ T cells by IDO1 was established as another important mechanism of immune evasion (Liu *et al*, 2009). Our finding of prognostic impact of IDO1 expression without correlation with the number of CD3^+^ or CD8^+^ lymphocytes supports such mechanism. In addition, the complex interaction between IDO1 and T lymphocytes in the tumour microenvironment makes it very difficult to detect the effect of IDO1 on T lymphocytes as merely a decrease in number. Moreover, the interplay in the tumour microenvironment becomes even more complicated if not only neoplastic epithelium, but also cells in the tumour stroma are taking into account as a possible source of IDO1 production. These cells contribute to anticancer immune reactions in hepatocellular carcinoma (Ishio *et al*, 2004). The intricate interaction between IDO1 and T lymphocytes was partly illustrated by our *in vitro* study on human colon carcinoma cell lines. We and others (Brandacher *et al*, 2006) found virtually no constitutive IDO1 expression in these cell lines; IDO1 expression, however, was induced upon IFN-*γ* stimulation. IFN-*γ* has been reported to be an effector cytokine released by tumour-associated antigen-specific T cells within the tumour microenvironment (Coussens and Werb, 2002). The inflammatory component of a neoplasm includes a diverse leukocyte population loaded with an assorted array of cytokines, cytotoxic mediators including reactive oxygen species, proteases, membrane-perforating agents and soluble mediators of cell killing such as TNF-*α* and IFNs (Kuper *et al*, 2000; Di Carlo *et al*, 2001; Dumont *et al*, 2008). Moreover, several inflammatory diseases increase the risk of developing cancer (Mantovani *et al*, 2008). Interestingly, high expression of IDO1 has also been observed in experimental murine colitis and in human inflammatory bowel disease, a condition that is associated with a significant increase in the risk of CRC (Wolf *et al*, 2004; Ferdinande *et al*, 2008; Hansen *et al*, 2009), further suggesting a role for IDO1 in the development and progression of CRC. In contrast, colorectal adenomas, dysplastic precursor lesions of CRC, show comparable IDO1 immunoreactivity as normal colonic mucosa (no staining or weak staining, unpublished results). Possibly, the absence of an extensive inflammatory reaction as seen in invasive cancer or inflammatory bowel disease tissue results in lower IDO1 expression levels (Cui *et al*, 2009).

Our study shows that IDO1 immunohistochemistry on pathological resection specimens of primary colorectal tumours can aid in making individualised therapeutic decisions. Indeed, stage-III CRC patients comprise a heterogeneous group of patients, where the need for adjuvant treatment is weighed against its possible side effects in patients with important comorbidity. Analysis of IDO1 expression at the tumour invasion front can be of additional value in the therapeutic decision-making process of pN1-staged patients. Our results also suggest that IDO1 blockade could be an effective therapeutic approach in selected CRC patients. In mouse models of breast cancer, IDO1 inhibition with 1-methyltryptophan had only limited therapeutic effects in monotherapy, but the antitumour activity of this compound was enhanced when it was given in combination with classical cytotoxic agents such as paclitaxel (Muller *et al*, 2005). Development of small molecule inhibitors or siRNA targeting the *IDO1* gene (Yen *et al*, 2009) result in promising new therapeutic options for IDO1 inhibition, but the efficacy in CRC remains to be clarified.

In conclusion, IDO1 is expressed in CRC cells upon IFN-*γ* stimulation, and IDO1 expression at the tumour invasion front, but not in the centre of the tumour, is an independent prognostic factor in the pT1-4N1Mx-staged CRC. Our results indicate that IDO1 is a reliable and promising prognostic indicator in CRC, which may provide a novel target for therapeutic intervention.

## Figures and Tables

**Table 1:** Clinical and histopathological patient data

**Features**	
Number of patients (%)	265 (100)
Age (years; (median (min–max))	70 (35–92)
Male/female ratio (%)	121/144 (46/54)
	
*Tumour stage*	
T0 (%)	1 (<1)
T1 (%)	50 (19)
T2 (%)	101 (38)
T3 (%)	110 (42)
Tx (%)	3 (1)
	
*Nodal status*	
N0 (%)	152 (57)
N1 (%)	78 (29)
N2 (%)	32 (12)
Nx (%)	3 (1)
	
Intact/defective MMR protein expression (%)	233/20 (92/8)

Abbreviation: MMR=mismatch repair.

Twelve cases could not be evaluated for MMR protein expression.

**Table 2:** Cox regression models for pT1-4N1Mx-staged colorectal cancer

**Model *P*-value**	**Prognostic features**	**b**	**e^b^**	***P*-value**
*(A) Overall survival* a				
0.0001	Age	0.051	1.053	0.0003
	T status	−0.042	0.959	0.879
	Mean IDO1 score in tumor front	0.441	1.554	0.001
				
*(B) Development of metachronous metastases* a				
0.00002	Male gender	−1.402	0.246	0.0002
	T status	0.459	1.582	0.175
	Mean IDO1 score in tumor front	0.432	1.540	0.018

Abbreviation: IDO1=indoleamine 2,3-dioxygenase 1.

a The equation at the basis of the Cox regression models is an exponential function of a linear combination of the included (quantitative, ordinal or binary) features, where *b* indicates each feature coefficient in the linear combination. The *e*^b^ value indicates that the risk of death or metastasis occurrence is increased by *e*^b^ % for patients belonging to the indicated category (in case of the binary feature) or per unit of the quantitative (such as mean IDO1 score) or ordinal (such as T status) feature. A positive *b* value thus means a negative impact on survival. The feature *P*-values are the levels of significance of the independent contributions of each feature to the model. If *P*<0.05, the feature is associated with a significant prognostic value, independent of all other features included in the model. The model *P*-value indicates the overall level of significance of the complete model.
